# Bilateral Orbital Myeloid Sarcomas: A Unique Presentation of Acute Myeloid Leukemia

**DOI:** 10.7759/cureus.27419

**Published:** 2022-07-28

**Authors:** Shane G Stephenson, Addison A Barchie, Hunaid N Rana, Todd B Standley, Maria S Figarola

**Affiliations:** 1 Department of Radiology, University of South Alabama College of Medicine, Mobile, USA; 2 Department of Pediatric Radiology, University of South Alabama College of Medicine, Mobile, USA

**Keywords:** brain mri, radiology, acute myeloid leukemia, granulocytic sarcoma, ocular chloroma, pediatrics

## Abstract

Myeloid sarcomas (MS) are solid manifestations of acute myeloid leukemia (AML) and are commonly present in children. These tumors can arise in many tissues including bone, soft tissue, or skin, and are commonly seen in the orbit. As practically all MS will, if left untreated, eventually present as AML, early diagnosis and initiation of treatment are imperative. We highlighted a case of bilateral orbital MS in a pediatric patient that presented concurrently with AML and the steps taken to diagnose and initiate treatment. Our case highlights the potentially occult presentation of AML as well as myeloid sarcoma and, therefore, the importance of swift workup and diagnosis. Epidemiology, radiographic features, diagnosis, and treatment for myeloid sarcoma and AML were discussed.

## Introduction

Myeloid sarcomas (MS) are extramedullary tumors composed of immature granulocytic cells. MS is often also referred to as granulocytic sarcomas (GS) or chloromas [1]. These tumors can develop at any age but are most often seen in children under 15 years of age with no sex predilection [2]. MS can occur in several locations such as bone, soft tissue, skin, lymph nodes, and kidney [3]. In the head and neck, the most commonly involved area is the orbit [2]. For orbital MS, the mean age of diagnosis is 8.8 years, and the median survival after diagnosis is 7.5 months [2,3]. The most common clinical presentation of orbital MS is rapidly progressing proptosis [4]. Other symptoms include redness and swelling of the eye unilaterally or bilaterally as well as blindness [3]. Interestingly, myeloid sarcoma has been identified as an extramedullary presentation of acute myeloid leukemia (AML), occurring in 2.5%-9.1% of patients with AML [1,5]. Additionally, orbital MS has been shown by several studies to occur in as many as 9.3%-36% of children with AML [6]. Less commonly, MS has been associated with other disorders of the bone marrow such as myelodysplastic syndrome, chronic myeloid leukemia, and other myeloproliferative disorders [1].

Orbital MS can appear at any point in the course of AML, sometimes even as the initial presenting symptom, prior to abnormal blood counts or bone marrow findings [2,6]. Orbital symptoms are more easily recognizable to parents, but primary orbital MS is often misdiagnosed by the pathologist. Most commonly, primary MS is misdiagnosed as large cell lymphoma but also as neuroblastoma, rhabdomyosarcoma, Ewing sarcoma, and carcinoma to name a few [1,3,5]. Correct diagnosis early on is imperative, because it changes the course of treatment significantly and has a dramatic effect on prognosis. Due to the very high rate (88%-100%) of transformation from MS to AML within a year when treated locally, systemic chemotherapy is recommended upon discovery of MS [1-3]. Therefore, it is important to recognize and accurately diagnose MS early and understand the treatment implications with leukemia. Hereby, we present a case of bilateral orbital MS in a child concurrently diagnosed with AML and discuss the proper diagnosis and management of these patients.

## Case presentation

An eight-year-old Caucasian male with no significant past medical history was admitted to the pediatric inpatient service following the discovery of pancytopenia by the patient’s primary care physician. The patient’s mother stated that he had a fever that resolved with acetaminophen three weeks prior to hospital admission. She also stated that his skin appeared paler than usual over this time frame. Additionally, she noticed that the patient’s right upper eyelid was swollen, which she assumed was allergic. However, loratadine failed to provide relief. The father stated that the patient had a single episode of epistaxis, intermittent thigh pain, and mild headaches over the last few weeks.

Initial laboratory studies were remarkable for a hemoglobin of 6.1 g/dL (reference range: 13.5-17.5 g/dL), reticulocyte count of 1% with high immature reticulocyte fraction indicating erythropoiesis, hematocrit of 17.5% (reference range: 41-53%), white blood cell (WBC) count of 1,190/mm^3^ (reference range: 4,500-11,000/mm^3^), and platelet count of 80,000/mm^3^ (reference range: 150,000-400,000/mm^3^). Additionally, there was an elevated prothrombin time (PT), partial thromboplastin time (PTT), international normalized ratio (INR), and lactate dehydrogenase (468 U/L, reference range: 45-90 U/L). A chest x-ray performed to rule out space occupying lesions was unrevealing.

The following morning, the patient received one unit of packed red blood cells for the treatment of symptomatic anemia; a blood sample was drawn for flow cytometry, and an abdominal ultrasound was performed. The ultrasound revealed hepatosplenomegaly with trace hepatic and perisplenic fluid and multiple prominent lymph nodes. The finding of hepatosplenomegaly in the context of pancytopenia was highly suggestive of a malignant process such as leukemia. Flow cytometry of peripheral blood revealed increased abnormal monocyte precursors, suggestive of a high-grade myeloid neoplasm. Later that day, a bone marrow biopsy revealed the diagnosis of AML of monoblastic differentiation with mild myelofibrosis.

**Figure 1:**
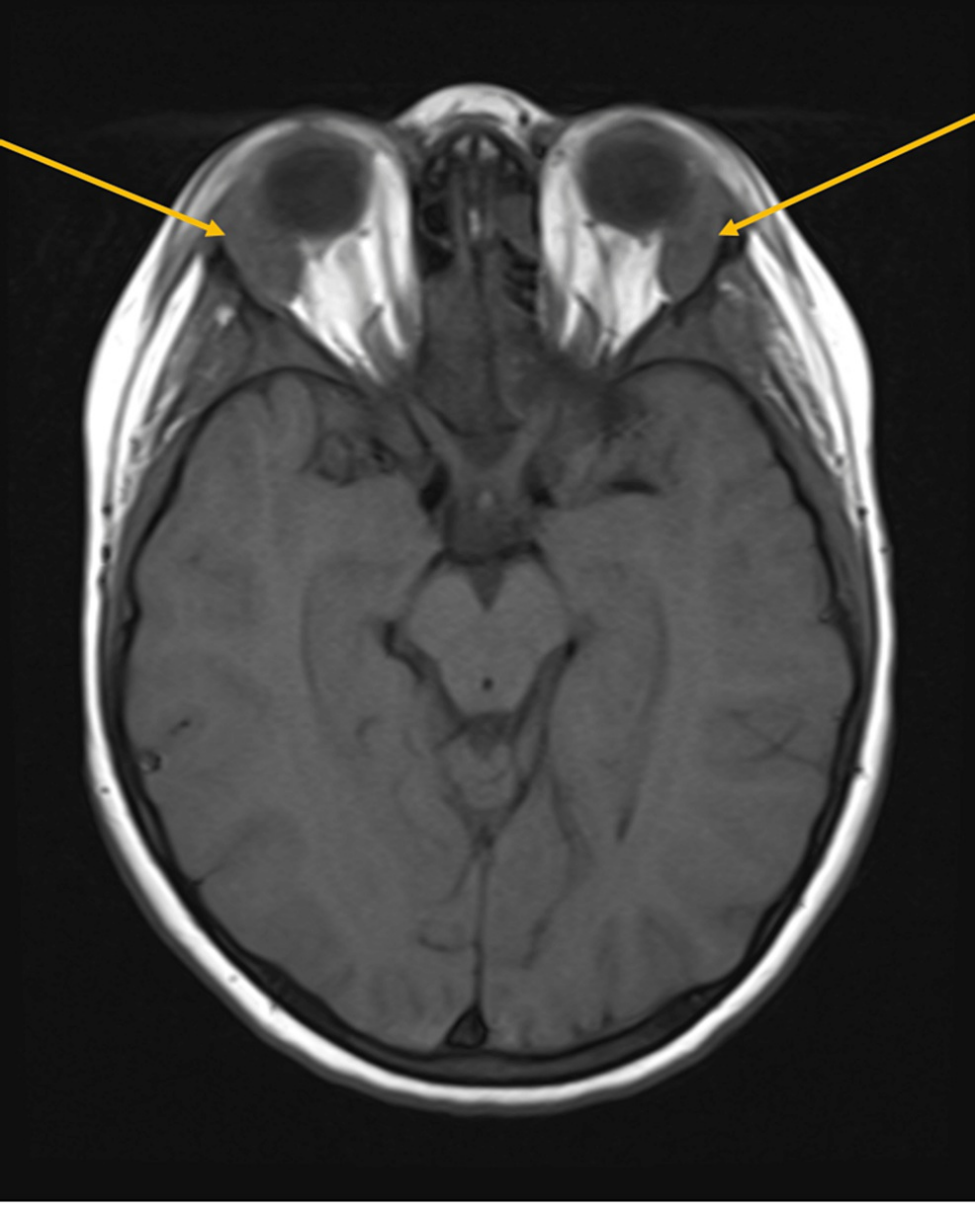
Axial T1-weighted MRI demonstrating intermediate signal of the orbital masses

**Figure 2:**
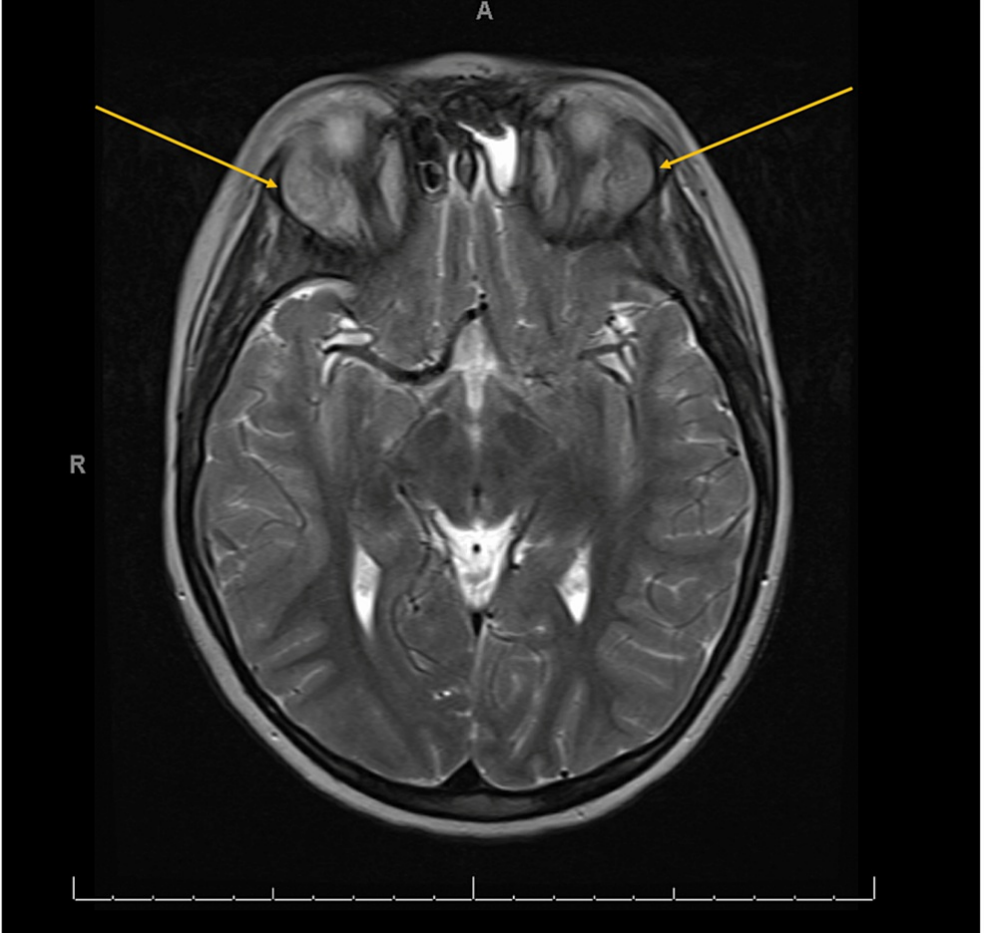
Axial T2-weighted MRI demonstrating intermediate to slightly low signal intensity of the orbital masses

**Figure 3:**
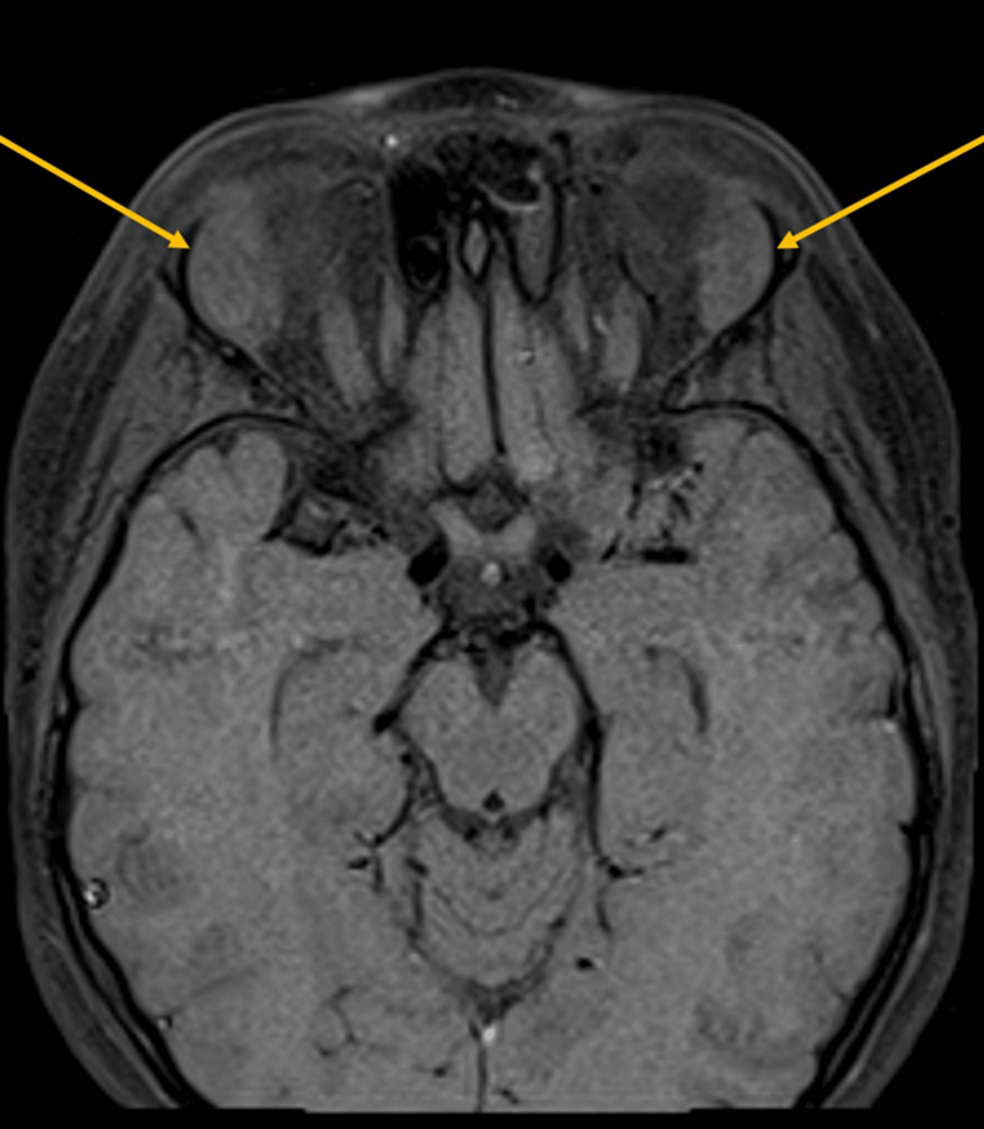
Axial MRI following gadolinium contrast demonstrating mild, homogenous contrast enhancement

**Figure 4:**
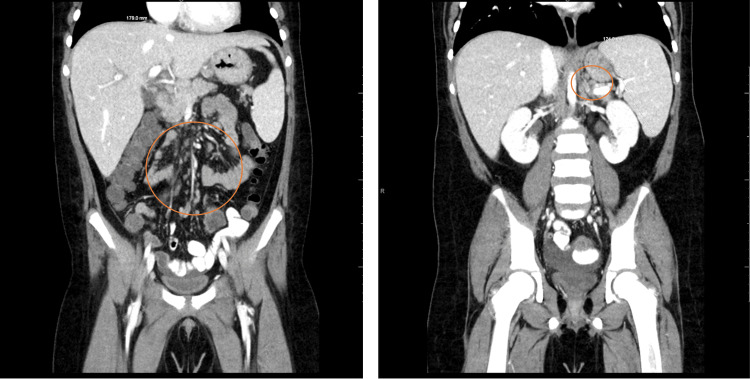
Coronal CT of the abdomen and pelvis demonstrating liver and spleen dimensions (hepatosplenomegaly) and numerous enlarged lymph nodes

Magnetic resonance imaging (MRI) of the head was performed to evaluate the right periorbital swelling. The images showed bilateral, ovoid-shaped, extraconal masses in the superior lateral aspect of the orbits with intermediate T1 signal (Figure 1), intermediate to slightly low T2 signal (Figure 2), and mildly increased short tau inversion recovery (STIR) signal. They demonstrated mild, homogenous post-contrast enhancement (Figure 3), and mild diffusion restriction. In the setting of a known clinical history of leukemia, these findings were suggestive of bilateral orbital MS. The following day, computed tomography (CT) of the abdomen and pelvis was performed and demonstrated hepatosplenomegaly with trace-free fluid in the pelvis and numerous enlarged lymph nodes in the upper abdomen (Figure 4). These findings were consistent with the diagnosis of AML.

Two days following admission, the patient was started on chemotherapy. He continued to be monitored for worsening periorbital symptoms over the following week, and induction of chemotherapy was tolerated well.

## Discussion

Orbital MS are extramedullary tumors that can appear at any age but are commonly present in children with 60% of cases occurring in children under the age of 15 years with a mean age of 8.8 [2,3]. These tumors, presenting clinically as red, swollen eyes, proptosis, or blindness, progress symptomatically very rapidly. However, these symptoms are not the greatest concern. Importantly, MS has been shown to be a harrowing sign of an even more serious concern: AML [3]. Among pediatric patients diagnosed with AML, 9.3%-36% will also present with orbital MS [5]. These MS can present at any point in the clinical progression of AML. They can be the initial presentation, they can present concurrently which is most common, or they can occur subsequent to the treatment of AML [6]. The importance of early diagnosis lies with the treatment plan. The recommendation from numerous sources is to begin immediate systemic chemotherapy upon diagnosis of MS as delayed or local treatment will most often lead to the development of clinically significant AML within the year [1,5].

This case emphasizes the importance of clinical suspicion for MS by physicians when young patients present with orbital symptoms. In this case, the diagnosis of bilateral orbital MS and AML was made within two days. However, the disease had already become systemic as represented by the symptoms of pancytopenia, hepatosplenomegaly, and prominent abdominal lymph nodes. In a similar case, a three-year-old male patient was initially misdiagnosed with acute bronchitis and anemia after presenting with pale skin, dry cough, and rhinorrhea. He was treated for a week before proptosis of the right eye suddenly developed. However, it took several more days, and a worsening condition before the correct diagnosis was reached [7]. These cases illustrate how difficult the diagnosis of MS can be. However, because MS often foreshadows the clinical presentation of AML, it can be used as a mechanism of early diagnosis and treatment. Therefore, in cases where signs of MS appear before clear evidence of leukemia, it is important to make an accurate, timely diagnosis. MS that appears first is sometimes misdiagnosed as malignant lymphoma, neuroblastoma, rhabdomyosarcoma, or carcinoma [1,3,5]. Additionally, when the diagnosis of leukemia is already known, MS can be overlooked as complications of leukemia such as infection, secondary tumors, or hemorrhage [8].

As radiologists are often the key players in making these differentiations, it is important to be aware of the key radiologic features of MS. The most common site of MS is the orbit, more specifically the subperiosteum of the roof of the orbit [2]. In order to distinguish MS from the complications of leukemia, it is necessary to use MR or CT imaging with contrast. These cranial CT and MR images should show homogeneous contrast enhancement in all studies [8]. MR sequences are the preferred imaging modality for the diagnosis of orbital MS, and T1, T2, and post-contrast studies should always be performed. On T1-weighted sequences, the tumors will appear slightly hyperintense to gray matter, muscle, and bone marrow. On T2-weighted sequences, they will appear isointense to white matter, muscle, and bone marrow. These tumors do not tend to cause the destruction of nearby bony structures and are usually fairly confined [9]. When suspected clinically, proper immunohistochemistry and hematological testing can be initiated to make a definitive diagnosis [5]. In cases where immunohistochemistry is not used, about 50% of diagnoses are missed. Therefore, a panel of CD43, lysozyme, myeloperoxidase (MPO), CD68, CD163, CD117, CD3, and CD20 is typically used and can correctly identify the majority of myeloid sarcomas [10]. Ultimately, this case demonstrates the importance of family education on symptoms to watch for, physician awareness of the key features of myeloid sarcoma, and physician awareness of the association between AML and myeloid sarcoma.

## Conclusions

We highlighted a case of bilateral orbital myeloid sarcomas in a pediatric patient, including the initial presentation, diagnosis, and treatment. Epidemiology, radiographic features, diagnosis, and need for treatment were discussed for patients with myeloid sarcoma and AML. The importance of clinical suspicion and rapid diagnosis was illustrated by the rapid evolution of disease seen in our case and many others.
